# Susceptibility to Alcohol Hangovers: The Association with Self-Reported Immune Status

**DOI:** 10.3390/ijerph15061286

**Published:** 2018-06-18

**Authors:** Aurora J. A. E. van de Loo, Marlou Mackus, Marith van Schrojenstein Lantman, Aletta D. Kraneveld, Karel A. Brookhuis, Johan Garssen, Andrew Scholey, Joris C. Verster

**Affiliations:** 1 Division of Pharmacology, Utrecht University, 3584CG Utrecht, The Netherlands; a.j.a.e.vandeloo@uu.nl (A.J.A.E.v.d.L.); M.Mackus@uu.nl (M.M.); m.vanschrojensteinlantman@students.uu.nl (M.v.S.L.); a.d.kraneveld@uu.nl (A.D.K.); j.garssen@uu.nl (J.G.); 2 Institute for Risk Assessment Sciences (IRAS), Utrecht University, 3584CM Utrecht, The Netherlands; 3 Faculty of Behavioral and Social Sciences, Groningen University, 9712TS Groningen, The Netherlands; k.a.brookhuis@rug.nl; 4 Nutricia Research, 3584CT Utrecht, The Netherlands; 5 Centre for Human Psychopharmacology, Swinburne University, Melbourne, VIC 3122, Australia; andrew@scholeylab.com

**Keywords:** alcohol, hangover, resistance, immune function

## Abstract

Increasing evidence points at a role for the immune system in the genesis of the alcohol hangover. This study investigated the association between self-reported immune function and experiencing hangovers. Dutch students aged 18 to 30 years old were invited to complete an online survey. Eighteen items on immune-related complaints were completed to assess self-reported immune function. Alcohol consumption in the past month (with respect to usual consumption and the occasion of heaviest drinking) was also recorded. Subjects with an estimated blood alcohol concentration (eBAC) of 0.18% or higher on their heaviest drinking occasion in the prior month were included in the analyses. Self-reported immune function was compared between drinkers with a hangover and those who claimed to be hangover resistant. In total, of 481 subjects (79.2% women) with a mean (SD) age of 21.1 (1.9) years old were included in the analysis. Of these, 83.3% (*n* = 400) reported having hangovers and 16.8% (*n* = 81) claimed to be hangover resistant. Drinkers with hangovers had significantly higher self-reported overall immune function scores when compared to hangover-resistant drinkers (mean ± SD = 10.5 ± 3.6 versus 13.1 ± 4.9, *p* = 0.0001), indicating a poorer immune status. In conclusion, experiencing alcohol hangovers is associated with significantly poorer self-reported immune function.

## 1. Introduction

The alcohol hangover refers to the combination of mental and physical symptoms experienced the day after a single episode of heavy drinking, starting when the blood alcohol concentration approaches zero [1]. Although most social drinkers experience a hangover the day following an evening of heavy drinking, some drinkers claim to be hangover resistant [2,3,4].

A recent study that directly compared social drinkers who experienced hangovers with those who claimed to be hangover resistant revealed that, in general, the characteristics of both groups were comparable. This similarity included their sensitivity to the effects of alcohol, as assessed with the Self-Rating of the Effects of alcohol (SRE) form [5]. Nevertheless, despite consuming the same, relatively high levels of alcohol, a group of drinkers still claimed to be hangover resistant. Identifying how hangover-resistant drinkers differ from those who do experience hangovers may help to elucidate the pathology of the alcohol hangover, an area that, to date, has received little research attention [6].

The immune system is one candidate mediator of the alcohol hangover. Studies have shown inflammatory effects after acute alcohol administration, such as significantly elevated serum cytokine levels 2 h after drinking [7]. It has been hypothesized that these inflammatory effects persist over longer time, and/or have an influence on the presence or severity of a next-day alcohol hangover [8].

This idea of the stimulation of the immune system being involved with the alcohol hangover has received some research attention. For example, in healthy male social drinkers, Kim et al. compared blood cytokine concentrations during the hangover state with those observed on an alcohol-free test day [9]. Blood samples were taken 13 h after drinking alcohol and during the placebo condition for measurement of cytokines following phytohemagglutinin stimulation of peripheral blood mononuclear cells. Concentrations of interleukin (IL)-10, IL-12, and interferon-gamma (IFN-γ) were significantly increased during the hangover state, whereas no significant differences were seen for concentrations of IL-1β, IL-4, IL-6, and tumor necrosis factor-alpha (TNF-α). Hangover severity scores were significantly positively correlated with blood cytokine concentrations of IFN-γ and IL-12, strengthening the idea that the immune system is involved in the pathology of the alcohol hangover.

Given this, it can be hypothesized that hangover-resistant drinkers may have a better-functioning immune system when compared to drinkers who do experience alcohol hangovers. The aim of the current study was to compare self-reported immune function of those who experience an alcohol hangover after heavy drinking with those drinkers who claim to be hangover resistant. It was hypothesized that those who experience hangovers had poorer self-reported immune function.

## 2. Materials and Methods

An online survey was designed using Survey Monkey and, via Facebook, Dutch students aged 18 to 30 years old were invited to participate. Written informed consent was obtained from all participants, and The University of Groningen Psychology Ethics Committee approved the study (16072-O).

Alcohol consumption in the prior month was recorded, with questions adapted from the Quick Drinking Screen [10]. By applying a modified Widmark equation [11], which takes into account weight and gender, the estimated blood alcohol concentration (eBAC) was calculated for the heaviest drinking occasion of the participants in the prior month. As the eBAC calculation includes information on gender, the amount of alcohol consumed, and the duration of drinking, for the current dataset it is the most integral alcohol-consumption variable to differentiate hangover-sensitive and hangover-resistant drinkers. Participants were asked whether or not they experienced a hangover during the past month, which allowed the creation of two groups: (1) the hangover-sensitive group; and (2) the hangover-resistant group.

Eighteen items from the Immune Function Questionnaire (IFQ) were completed to examine past year self-reported immune status [12]. The items included sore throat, headache, flu, runny nose, coughing, cold sores, mild fever, warts, pneumonia, bronchitis, sinusitis, sudden high fever, ear infection, diarrhea, meningitis, eye infection, sepsis, and long-healing injuries. Each item was rated on a 5-point Likert-type scale (Never, Once or twice, Occasionally, Regularly, Frequently), yielding scores ranging from 0 to 4. In addition, an overall immune function score was computed, ranging from 0 to 72, with higher scores reflecting poorer immune function. Previous research showed that IFQ scores correlated significantly with perceived health, a one-item immune function score, mental resilience, and autistic traits [13,14].

The data were analyzed using SPSS, version 24. Associations between self-reported immune function and drinking parameters were explored using Pearson’s correlations. Depending on the distribution of the data either analyses of variance (ANOVA) or a nonparametric, independent-sample Mann–Whitney U test was used to compare the two groups. Differences were significant if *p* < 0.05.

To be included in the statistical analyses, participants had to have an eBAC of at least 0.18% on their heaviest drinking occasion in the prior month. This cut-off was chosen based on the average eBAC of 0.18% which was recently observed in a naturalistic study comparing hangover-sensitive and hangover-resistant drinkers [5]. Using this relative high eBAC ensured that participants had consumed a sufficient amount of alcohol to experience a hangover per se [4]. To infer whether the achieved eBAC has an impact on levels of self-reported immune function, overall immune function scores of hangover-sensitive and hangover-resistant drinkers were compared across the full range of eBAC scores, with increments of 0.01 BAC%, including an eBAC of at least 0.11%, i.e., the lower limit BAC for provoking hangovers suggested in 2010 by the Alcohol Hangover Research Group [15].

## 3. Results

**Figure 1:**
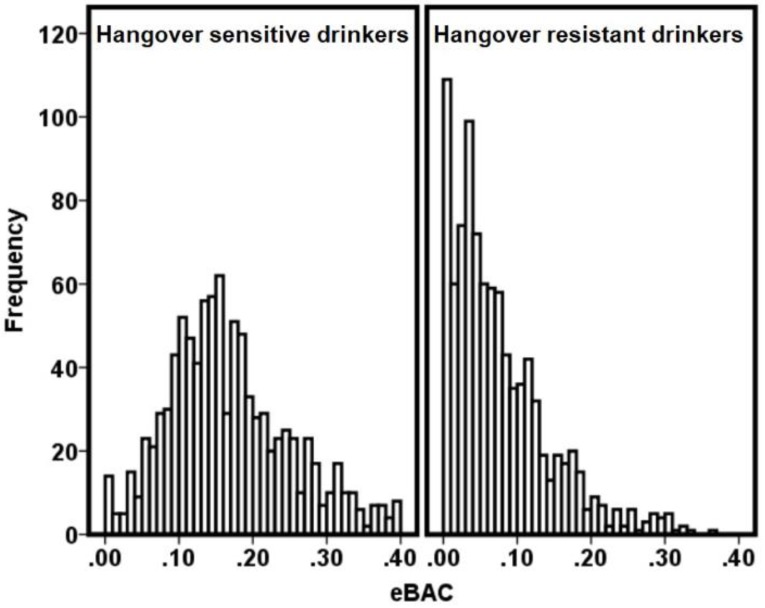
The number of hangover-sensitive and hangover-resistant drinkers at different eBAC levels. Abbreviation: eBAC = estimated blood alcohol concentration.

The survey was completed by *n* = 2295 subjects, of whom 83.4% were women (*n* = 1911). The vast majority of them reported past month alcohol consumption (*n* = 1937, 84.4%). Approximately half of these participants (51.1%) had experienced a hangover during the prior month. The overall self-reported immune function did not significantly correlate with the usual number of drinks consumed on a regular drinking day (*r* = 0.028, *p* = 0.227). Significant associations were found between the overall self-reported immune function score and the maximum number of drinks consumed on the heaviest drinking occasion in the prior month (*r* = 0.071, *p* = 0.003) and prior year (*r* = 0.067, *p* = 0.004). Figure 1 summarizes the distribution of the number of participants that were categorized as hangover sensitive and hangover resistant.

It is evident from Figure 1 that most drinkers who were categorized as hangover resistant had an eBAC below 0.11%. Also, with higher eBAC levels, the number of subjects that reported not having a hangover in the past month declined steadily.

### 3.1. eBAC Cut-Off of at Least 0.18%

In total, 481 participants (*n* = 100 men, *n* = 381 women) had an eBAC of at least 0.18% on their heaviest drinking occasion in the prior month and were included in the statistical analyses. During the past month, *n* = 400 (83.2%) of drinkers had a hangover (i.e., the hangover-sensitive group), whereas *n* = 81 (16.8%) reported no hangover (i.e., the hangover-resistant group). Their demographics are summarized in Table 1.

The two groups differed significantly in age of regular alcohol consumption, the number of drinks on average occasions, drinking days and number of days drunk of the past month, number of drinks on the heaviest occasion, and duration of alcohol consumption (hours). However, there was no significant difference in eBAC on the heaviest drinking occasion.

For drinkers with an eBAC of at least 0.18% on their heaviest drinking occasion in the prior month, overall self-reported immune function scores did not significantly correlate with the usual number of drinks consumed on a regular drinking day (*r* = 0.034, *p* = 0.461), nor the maximum number of drinks consumed on their heaviest drinking occasion in the prior month (*r* = 0.003, *p* = 0.949) and prior year (*r* = 0.026, *p* = 0.583).

Data on immune function are summarized in Table 2. Relative to hangover-resistant drinkers, those who reported a hangover had a significantly higher mean ± SD overall self-reported immune function score (10.5 ± 3.6 versus 13.1 ± 4.9, *p* = 0.0001), indicating a poorer immune status. For those with a hangover, significantly higher scores were reported on the individual items diarrhea (*p* = 0.008), sinusitis (*p* = 0.008), cough (*p* = 0.002), headache (*p* = 0.0001), and sore throat (*p* = 0.0001).

### 3.2. Other eBAC Cut-Off Levels

**Figure 2:**
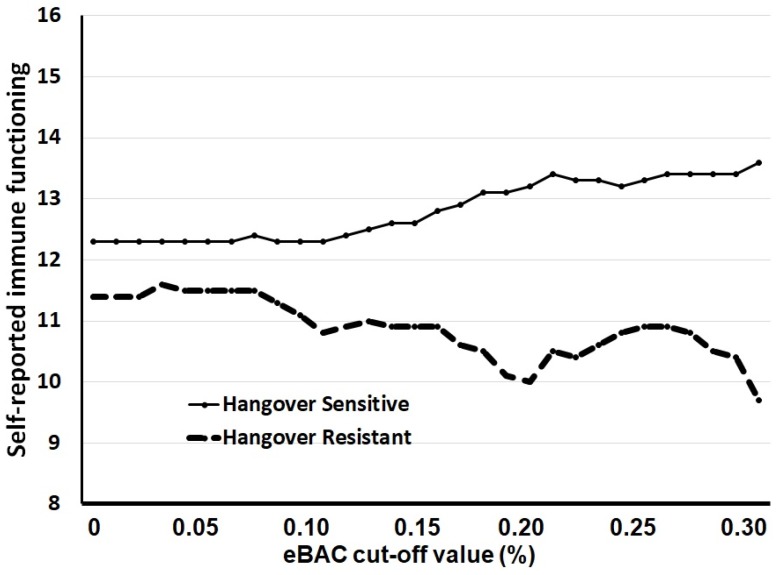
Immune function across eBAC cut-off levels. Abbreviations: eBAC = estimated blood alcohol concentration. For each eBAC cut-off level, participants were allocated to either the hangover-sensitive or hangover-resistant group, and their overall self-reported immune function score was computed.

The same analyses were conducted for other eBAC cut-off values up to 0.30%, with increments of 0.01%. For each eBAC cut-off value it was determined whether drinkers belonged to the hangover-sensitive or hangover-resistant group. Then, the overall self-reported immune function scores of the two groups were compared. The overall self-reported immune function scores of the hangover group and hangover-resistant group for each eBAC cut-off are summarized in Figure 2. For each eBAC cut-off value, the hangover-sensitive group reported a significantly higher overall self-reported immune function score than the hangover-resistant group.

It is important to note that for eBAC cut-off values up to 0.18% the hangover-sensitive group consumed significantly more frequent and greater amounts of alcohol and achieved significantly higher eBACs on their heaviest drinking occasion in the prior month. Given these significantly different average eBACs, a direct comparison of the immune status between hangover-sensitive and resistant-group is hard to interpret. For this reason, we choose not to present specific data for the eBAC 0.11% cut-off level, i.e., the lower limit BAC for provoking hangovers suggested in 2010 by the Alcohol Hangover Research Group [15]. Although the observed differences in immune status at eBAC 0.11% are comparable to those seen at eBAC 0.18%, the impact of the observed difference in quantity and pace of drinking and average eBAC (0.21% for hangover-sensitive and 0.18% for hangover-resistant drinkers) cannot be ruled out. With eBAC cut-off values higher than 0.18%, the achieved eBACs on the heaviest drinking occasion in the prior month did not significantly differ between the groups; however, usual alcohol intake (quantity and frequency) and alcohol consumption on the heaviest drinking occasion in the prior month remained significantly higher for the hangover group up to an eBAC cut-off value of 0.30%. This should be taken into account when interpreting Figure 2.

## 4. Discussion

The current analyses confirmed our hypothesis that drinkers who experience hangovers report significantly poorer immune function compared to hangover-resistant drinkers.

Previous research has shown that alcohol consumption provokes an immune response directly after the start of drinking [7], as well as immunological responses (cytokine concentration changes in saliva) during the alcohol hangover state [9]. These are however direct and next-day effects of alcohol, while in the current paper we observed that the usual level of immune function is different between hangover-sensitive and hangover-resistant drinkers. Although this observation may be related to hangover susceptibility, there are no clinical studies available that address the issue of causation. That is, based on the current data one cannot conclude whether a poorer immune status makes a drinker more susceptible to having alcohol hangovers, or alternatively, that having alcohol hangovers results in a poorer immune status. This could be a topic of future more detailed prospective studies for which our evidence sets the basis.

There are, however, a number of important issues that should be taken into account when interpreting the data. First of all, overall self-reported immune function scores can range from 0 to 72. As is evident from Figure 2, across eBAC cut-off levels the observed overall immune function scores are relatively low, suggesting both groups have relatively well-functioning immune systems. This is not surprising, as we assume that the target population of students aged 18 to 30 years old predominantly consists of healthy subjects. Second, Figure 2 also illustrates that, while statistically significant, the magnitude of the differences between the hangover-sensitive and hangover-resistant group is relatively small (≤4%) given the possible outcome range of 0 to 72. It should be taken into account when interpreting Figure 2 that only for eBAC levels of 0.18% and higher, the actual average eBAC of the hangover-sensitive and hangover-resistant group did not significantly differ. Third, there is consensus among the Alcohol Hangover Research Group members that eBACs below 0.11% are unlikely to provoke a hangover [15]. Hence, claiming hangover resistance at these drinking levels has little relevance. Fourth, the majority of the participants were female. This should not be a major concern, as previous research suggests that gender is not associated with incidence of hangover resistance. For example, Kruisselbrink et al. reported that, above eBACs of 0.08%, no significant gender differences were observed in the percentage of drinkers that were hangover-negative over their lifetimes (5.8% of female and 5.1% of male drinkers) [4]. Finally, the collected data were self-reported, which may introduce the risk of recall bias and can result in under- or overreporting [16]. However, the survey was anonymous, which reduces the risk of recall bias. Also, there is no reason to assume that possible recall bias effects would differentially affect hangover-sensitive and hangover-resistant drinkers.

In part, the observed difference can be explained by higher alcohol consumption levels (both on regular and heaviest drinking occasions) of the hangover-sensitive group.

Estimated BAC is a better differentiator between hangover-sensitive drinkers and hangover-resistant drinkers than the number of alcoholic drinks consumed on the heaviest drinking occasion, as the duration of drinking has an impact on intoxication levels and its functional consequences. However, if usual levels of alcohol consumption and the frequency of drinking are higher in one group compared to the other, this may have an impact on overall immune function, irrespective of the amounts consumed on the heaviest drinking occasion. On the other hand, when applying the eBAC cut-off value of 0.18%, associations between usual and maximum alcohol consumption levels did not correlate significantly with overall self-reported immune function scores. In other words, at this drinking level the absolute amount of alcohol consumed is not significantly related to self-reported immune status. The absence of this association may however be an independent observation from whether of not having hangovers is related to immune status.

Future research should examine the more direct immunological changes during alcohol intoxication and the subsequent hangover state. Preferably, these would include objective measures of immune status, such as cytokine concentration in blood and saliva, as well as microbiome and gut leakage after alcohol intoxication. This way, hangover-sensitive drinkers and hangover-resistant drinkers can be compared in a controlled setting. Such a setting would also allow pace and quantity of alcohol consumption to be matched across groups. Also, collecting information on hangover frequency per month and their severity can be important to further elucidate the role of the immune system in alcohol hangovers.

## 5. Conclusions

Notwithstanding the identified limitations of the current study, a consistent and statistically significant difference in overall self-reported immune function was observed across eBAC cut-off levels, implying that hangover-sensitive drinkers, as compared to hangover-resistant drinkers, have a poorer self-reported immune function.

## Figures and Tables

**Table 1:** Demographics of hangover-sensitive drinkers and hangover-resistant drinkers after achieving an estimated BAC of at least 0.18% during their heaviest drinking occasion in the prior month. Significant differences (*p* < 0.05) between the groups are indicated with *. Abbreviations: BMI = body mass index, eBAC = estimated blood alcohol concentration.

	Hangover Sensitive	Hangover Resistant	
	*n* = 400	*n* = 81	
	Mean (SD)	Mean (SD)	*p*-Value
Age (years)	21.1 (1.9)	20.7 (2.0)	0.047 *
BMI (kg/m^2^)	22.3 (2.8)	22.3 (3.3)	0.992
Age of regular alcohol consumption	16.3 (1.3)	16.6 (1.7)	0.045 *
Number of drinks per average occasion	8.5 (4.5)	7.0 (3.5)	0.004 *
Number of drinking days in the past month	11.5 (6.6)	8.9 (7.0)	0.001 *
Number of days drunk in the past month	4.7 (3.6)	2.3 (2.2)	0.0001 *
Number of drinks consumed on the heaviest occasion	15.6 (5.5)	13.8 (4.4)	0.006 *
Duration of alcohol consumption (h)	6.8 (2.4)	5.9 (2.1)	0.001 *
eBAC (%) on heaviest occasion	0.27 (0.1)	0.26 (0.1)	0.123

**Table 2:** Self-reported immune function of hangover-sensitive drinkers and hangover-resistant drinkers after achieving an estimated BAC of at least 0.18% during the heaviest drinking occasion in the prior month. Data was compared using an independent-sample Mann–Whitney U test. Significant differences (*p* < 0.05) between the groups are indicated with *.

	Hangover Sensitive	Hangover Resistant	
	*n* = 400	*n* = 81	
	Mean (SD)	Mean (SD)	*p*-Value
Overall score	13.1 (4.9)	10.5 (3.6)	0.0001 *
Headache	2.1 (0.9)	1.7 (0.9)	0.0001 *
Runny nose	2.0 (1.0)	1.8 (0.9)	0.088
Coughing	2.0 (1.0)	1.6 (0.9)	0.002 *
Sore throat	1.7 (0.9)	1.3 (0.8)	0.0001 *
Diarrhea	1.4 (1.0)	1.1 (0.9)	0.008 *
Flu	0.7 (0.7)	0.5 (0.6)	0.254
Mild fever	0.7 (0.7)	0.6 (0.6)	0.953
Sinusitis	0.3 (0.6)	0.1 (0.3)	0.008 *
Cold sores	0.3 (0.7)	0.3 (0.6)	0.626
Long-healing injuries	0.3 (0.7)	0.3 (0.6)	0.475
Ear infection	0.2 (0.6)	0.1 (0.3)	0.194
Warts	0.2 (0.6)	0.1 (0.4)	0.092
Eye infection	0.2 (0.5)	0.1 (0.4)	0.482
Bronchitis	0.1 (0.4)	0.1 (0.3)	0.680
Sudden high fever	0.1 (0.3)	0.1 (0.3)	0.204
Sepsis	0.1 (0.5)	0.1 (0.3)	0.088
Pneumonia	0.0 (0.2)	0.1 (0.2)	0.396
Meningitis	0.0 (0.0)	0.0 (0.0)	1.000
